# Complete Communicating Tubular Oesophageal Duplication in an Adolescent Presenting With Haematemesis and Long-Standing Dysphagia

**DOI:** 10.7759/cureus.113429

**Published:** 2026-07-27

**Authors:** Mehdi Zouaoui, Mohamed aymane Assili, Mounir El Bied, Mohamed Ayez, Youssef Hnach, Mbarek Azouaoui, Nourdin Aqodad

**Affiliations:** 1 Department of Hepato-Gastroenterology, Mohammed VI University Hospital, Agadir, MAR; 2 Faculty of Medicine and Pharmacy of Agadir, Ibn Zohr University, Agadir, MAR; 3 Department of Radiology, Mohammed VI University Hospital, Agadir, MAR; 4 Department of Hepato-Gastroenterology, Oued Eddahab Military Hospital, Agadir, MAR

**Keywords:** communicating tubular duplication, double oesophagus, dysphagia, haematemesis, oesophageal duplication

## Abstract

Complete communicating tubular oesophageal duplication is a rare congenital foregut anomaly that may remain undiagnosed until adolescence because symptoms can be intermittent. A 17-year-old boy with long-standing intermittent solid-food dysphagia presented with moderate haematemesis. Upper gastrointestinal endoscopy identified an ulcerated inflammatory stricture 26 cm from the incisors, an accessory orifice, and a diverticulum-like opening. Biopsies showed ulcerative and regenerative changes with glandular and intestinal metaplasia without dysplasia. Barium oesophagography and cervicothoracoabdominal computed tomography demonstrated chronic oesophageal dilatation and an anterior tubular structure running parallel to the thoracic oesophagus, with multiple communications to the native lumen and distal drainage towards the gastric cardia, without a tracheobronchial fistula. Repeat endoscopy with a paediatric gastroscope confirmed an accessory channel lined by oesophageal-type mucosa and draining into the stomach alongside a stenotic native lumen. Complete communicating tubular oesophageal duplication should be considered in patients with long-standing dysphagia and an accessory oesophageal opening. Diagnosis requires complementary endoscopic and radiological assessment, and management should be individualised through multidisciplinary review.

## Introduction

Oesophageal duplications belong to the spectrum of congenital foregut duplications. They are rare and most often present as cystic, non-communicating lesions adjacent to the thoracic oesophagus. The communicating tubular variant is considerably less common and consists of an elongated duplicated channel lined by gastrointestinal mucosa, sometimes sharing a common wall with the native oesophagus and communicating with it through one or more orifices [1,2]. In this report, "complete" refers to an accessory channel that communicates proximally with the native oesophagus and continues distally into the stomach, forming two patent oesophageal pathways, whereas a partial duplication is confined to only part of the oesophageal length.

The clinical presentation depends on the site, size, and communicating nature of the duplication and on associated complications. Symptomatic forms may manifest as dysphagia, chest or epigastric pain, vomiting, recurrent respiratory infections, fistulisation, gastrointestinal bleeding, or, rarely, malignant transformation [1,3,4]. In the present case, the intermittent and relatively well-tolerated nature of the dysphagia, together with the rarity of the anomaly, may have contributed to the delayed diagnosis.

We report a complete communicating tubular oesophageal duplication presenting with haematemesis in an adolescent with solid-food dysphagia since childhood. This report describes the endoscopic and radiological features that established the diagnosis and discusses the therapeutic implications of this rare malformation.

## Case presentation

A 17-year-old boy presented after two episodes of haematemesis. The bleeding volume was considered moderate clinically, but could not be reliably quantified. The episodes were associated with mild epigastric pain, without melena or dizziness. He reported no previous gastrointestinal bleeding. His medical history was notable for intermittent solid-food dysphagia since childhood, which had never been investigated. He had no other relevant medical history.

The dysphagia had persisted intermittently without chronic cough, aspiration, recurrent pneumonia, or other extra-digestive manifestations. His general condition was preserved (body mass index: 22 kg/m²; no growth retardation), and the general and abdominal examinations were unremarkable. On admission, he was haemodynamically stable and afebrile. The available vital signs and initial laboratory findings are summarised in Table 1.

**Table 1:** Vital signs and initial laboratory findings at admission Note: Reference intervals for biochemical tests are those of the reporting laboratory. Reference intervals for vital signs, complete blood count, and coagulation parameters correspond to the institution’s age-specific ranges. °C, degrees Celsius; mmHg, millimetres of mercury; beats/min, beats per minute; %, percent; g/dL, grams per decilitre; ×10⁹/L, 10⁹ cells per litre; mg/L, milligrams per litre; g/L, grams per litre; mmol/L, millimoles per litre; U/L, units per litre

Parameter	Patient value	Unit	Reference range
Body temperature	36.8	°C	36.0-37.5
Blood pressure	115/70	mmHg	90-119/60-79
Heart rate	82	beats/min	60-100
Peripheral oxygen saturation on room air	98	%	95-100
Haemoglobin	13.6	g/dL	13.0-17.0
Platelet count	268	×10⁹/L	150-400
White blood cell count	6.8	×10⁹/L	4.0-10.0
Prothrombin activity	96	%	70-100
International normalised ratio	1.02	Dimensionless	0.8-1.2
C-reactive protein	3.1	mg/L	<5
Urea	0.24	g/L	0.17-0.49
Serum creatinine	6.8	mg/L	6.7-11.7
Sodium	139	mmol/L	136-145
Potassium	4.2	mmol/L	3.5-5.1
Chloride	106	mmol/L	98-107
Aspartate aminotransferase	17	U/L	<50
Alanine aminotransferase	10	U/L	<50
Gamma-glutamyl transferase	19	U/L	<60
Total bilirubin	3.9	mg/L	0-10
Indirect bilirubin	1.9	mg/L	<7
Direct bilirubin	2	mg/L	<2
Alkaline phosphatase	72	U/L	40-150
Serum albumin	34.6	g/L	34-50

Initial management was conservative and comprised fasting, peripheral venous access, clinical and haemodynamic monitoring, and intravenous proton pump inhibitor therapy. No transfusion was required because the patient remained haemodynamically stable throughout 72 hours of inpatient monitoring.

Initial endoscopy

**Figure 1:**
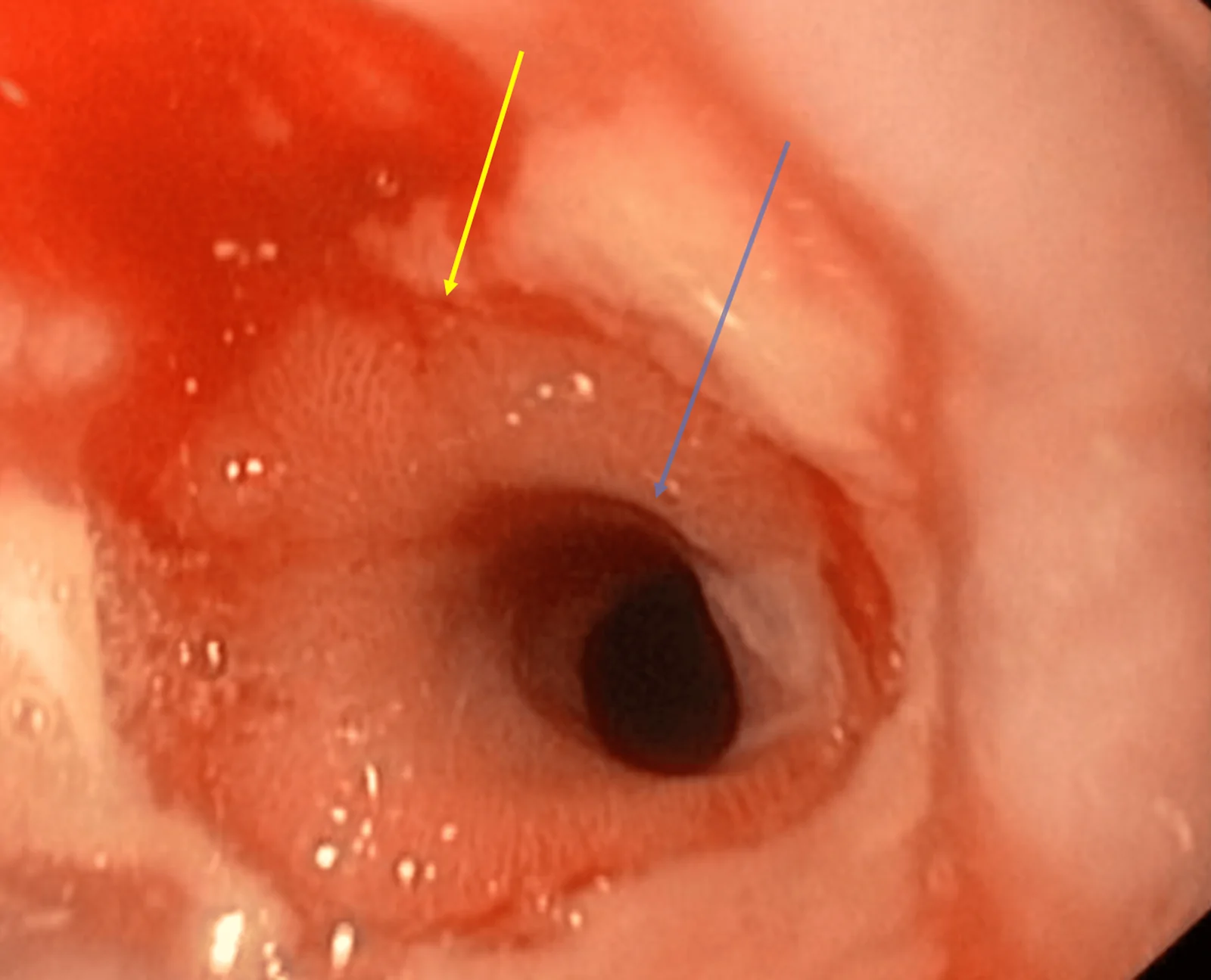
Endoscopic view of the oesophageal stricture located 26 cm from the incisors The blue arrow indicates the tight inflammatory stricture with a markedly narrowed residual lumen, while the yellow arrow indicates the surrounding congestive, oedematous, and focally ulcerated mucosa. The stricture could not be traversed with a standard adult gastroscope.

A first upper gastrointestinal endoscopy, performed during the evaluation of haematemesis, demonstrated an oesophageal stricture 26 cm from the incisors. The stricture was inflammatory and ulcerated and could not be traversed with a standard adult gastroscope (Figure 1). Immediately proximal to the stricture, an accessory orifice suggestive of a fistulous communication and a diverticulum-like outpouching were identified. The cardia and stomach could not be assessed because the stricture was not traversable. Biopsies from the stricture showed congestive mucosa with ulcerative and regenerative changes, glandular and intestinal metaplasia, and no dysplasia or malignancy.

Barium study

**Figure 2:**
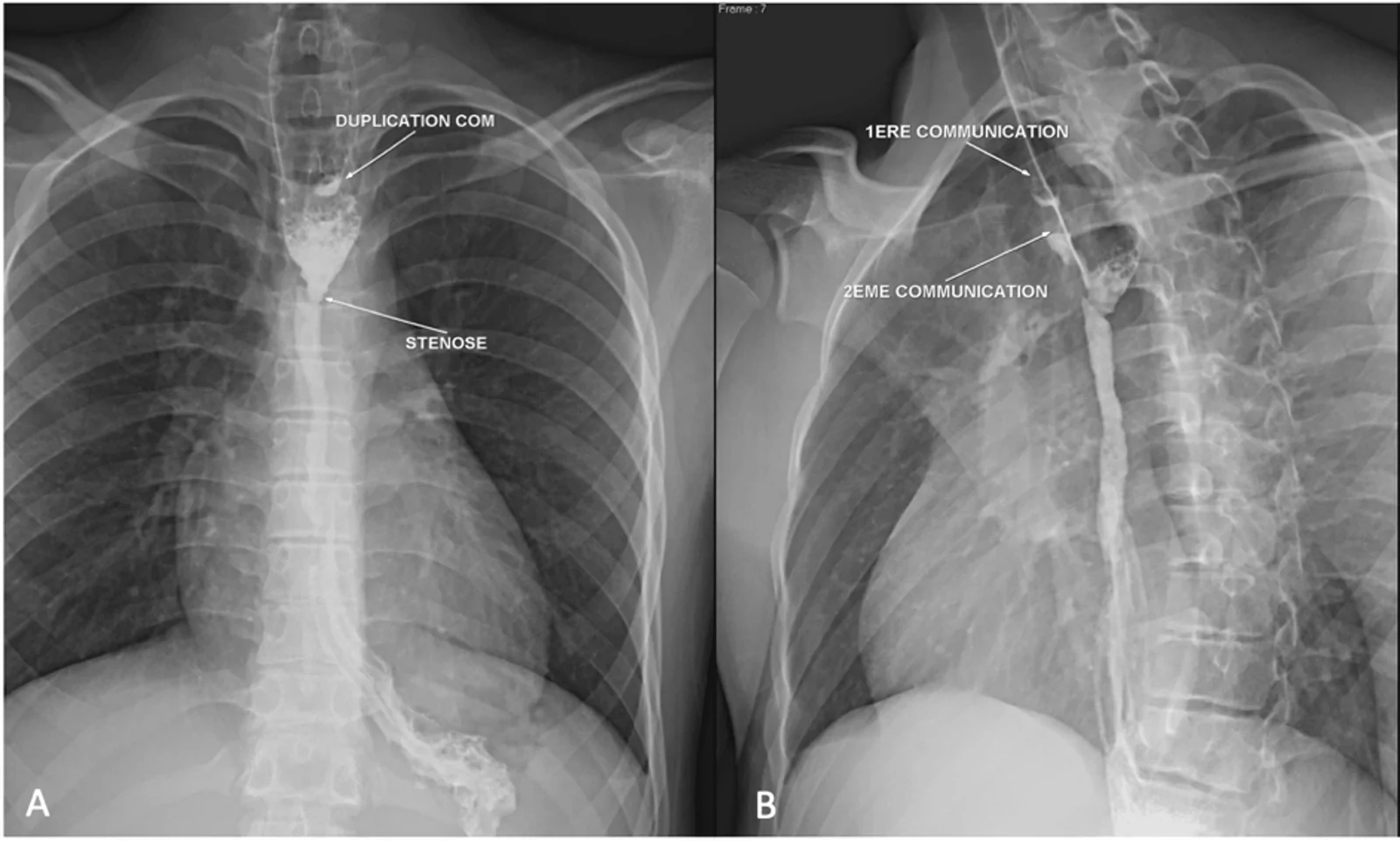
Barium study: frontal (A) and oblique lateral (B) projections A tapered stenosis of the middle third of the oesophagus is associated with moderate upstream dilatation. In the upper thoracic oesophagus, a small diverticulum-like outpouching (approximately 7 × 11 mm) communicates with the lumen at the T3 level and appears to be blind-ending distally. A second tubular contrast-filled channel at the T4 level runs parallel to the native lumen, communicates with the upper third of the oesophagus through two visible orifices, and extends to the gastric cardia, supporting the diagnosis of complete communicating tubular oesophageal duplication. No contrast passage into the tracheobronchial tree was observed.

**Figure 3:**
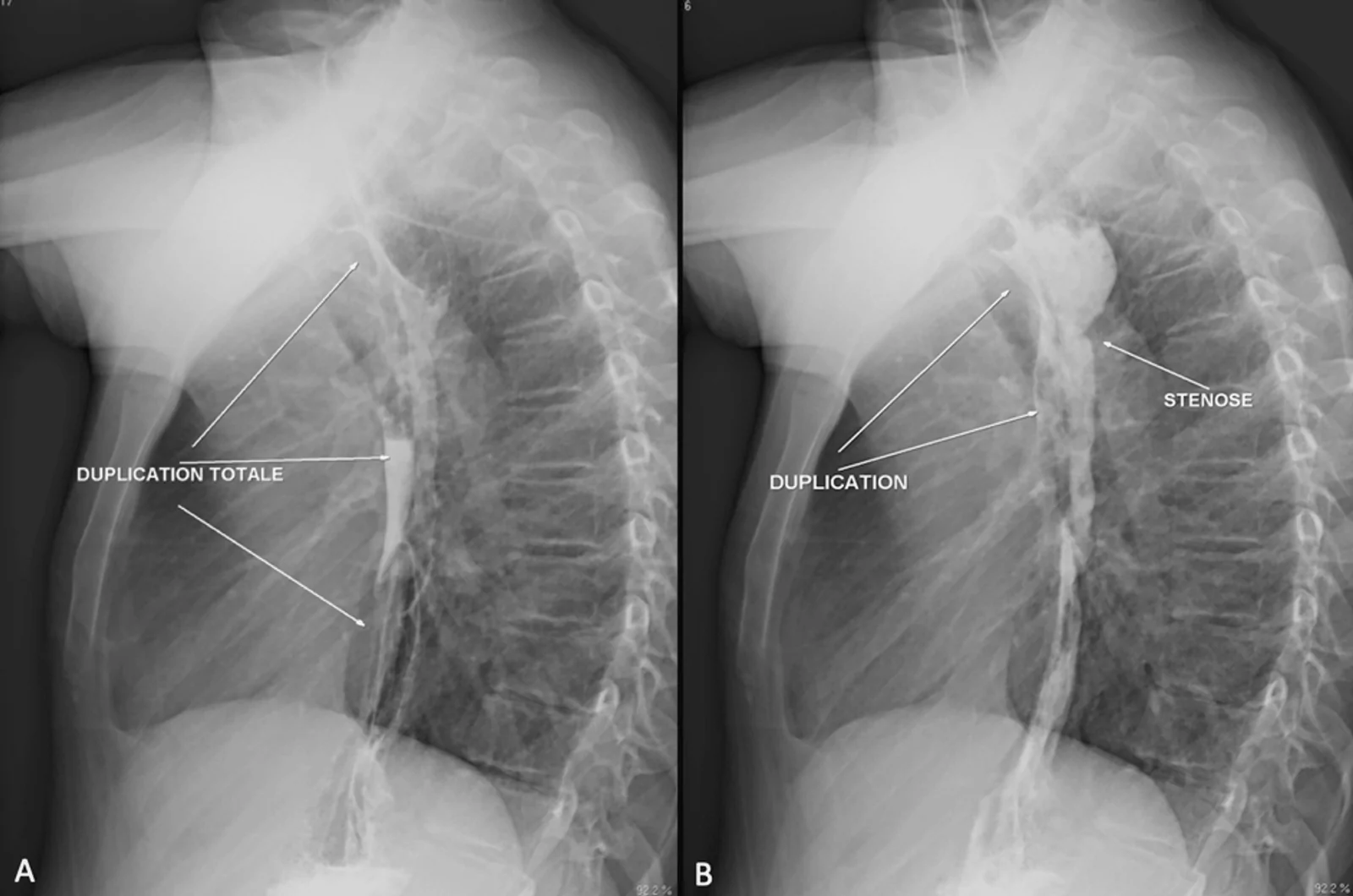
Barium study: lateral projections obtained during different phases of oesophageal opacification (A, B) A long contrast-filled tubular channel runs parallel to the native lumen, consistent with complete communicating tubular oesophageal duplication. The duplicated channel extends from the upper thoracic oesophagus to the distal oesophagus and gastric cardia and communicates with the main lumen at multiple levels. A tapered stenosis of the middle third of the native oesophagus is associated with upstream contrast stasis and moderate dilatation. The tracheobronchial tree is not opacified.

Given the combination of an oesophageal stricture, an accessory orifice, and a diverticulum-like opening, barium oesophagography and cervicothoracoabdominal computed tomography (CT) were performed. The barium study demonstrated a small diverticulum-like outpouching that appeared blind-ending distally, measured 7 × 11 mm, and communicated with the upper third of the oesophagus at the T3 level. A second tubular channel arose at the T4 level, ran parallel to the native oesophageal lumen, communicated with the upper third of the oesophagus, and extended towards the gastric cardia; this appearance was compatible with a complete communicating tubular oesophageal duplication. A tapered stenosis was centred on the middle third of the oesophagus, with moderate upstream dilatation. No communication with the tracheobronchial tree was demonstrated. A hiatus hernia and gastro-oesophageal reflux were also noted (Figures 2-3).

Computed tomography

**Figure 4:**
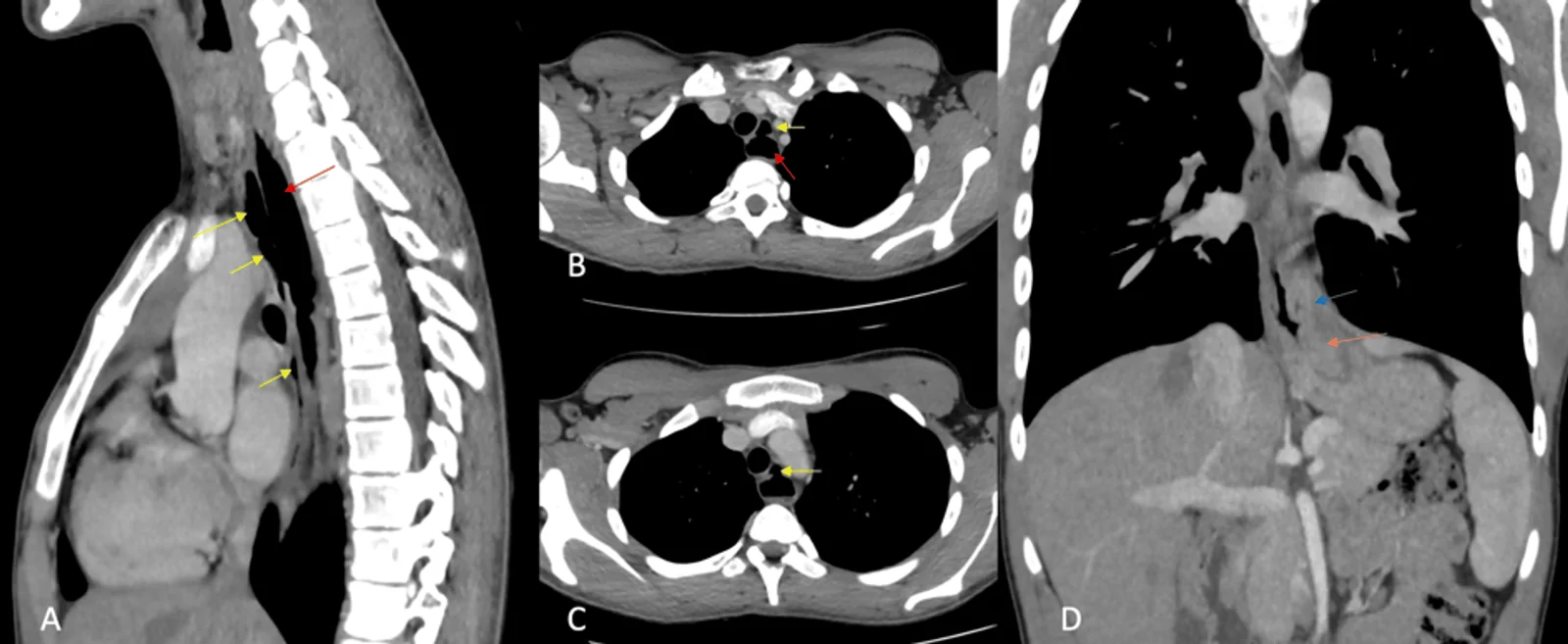
Contrast-enhanced CT acquired during the venous phase, with sagittal (A), axial (B, C), and coronal (D) reformations There is diffuse dilatation of the oesophagus, predominantly in the upper and middle thirds (red arrows), in the setting of a hiatal hernia (orange arrow). An air-filled structure lies anterior to the thoracic oesophagus, demonstrating three communications with the lumen in the upper third and two in the lower third (yellow arrows). This tubular structure shows mucosal contrast enhancement. There is mild, regular, presumed inflammatory thickening of the cardiofundic junction (10 mm) and the distal oesophageal wall (8.4 mm) (blue arrow). No abnormality of the mediastinal fat or cervical or thoracic lymphadenopathy is observed.

Cervicothoracoabdominal CT, performed before and after intravenous iodinated contrast administration, confirmed dilatation of the thoracic oesophagus, predominantly in the upper two-thirds. An air-containing tubular structure was present anterior and adjacent to the thoracic oesophagus, with several communications to the main oesophageal lumen. Its wall was enhanced after contrast administration; this finding was compatible with a mucosal lining. An oesophageal-type mucosal surface was subsequently observed endoscopically, but no histological confirmation was obtained. On contrast-enhanced CT, the maximal apparent wall thickness measured 10 mm at the cardiofundic junction and 8.4 mm in the distal oesophagus. The smooth, regular appearance, absence of a focal mass or lymphadenopathy, and preserved surrounding fat favoured an inflammatory rather than neoplastic process. The mediastinal fat was normal, with no cervical or thoracic lymphadenopathy, pleuropericardial involvement, or pulmonary abnormality. Abdominal evaluation showed no gastric or duodenal duplication, abdominal mass, or deep lymphadenopathy (Figure 4).

Characterisation endoscopy

**Figure 5:**
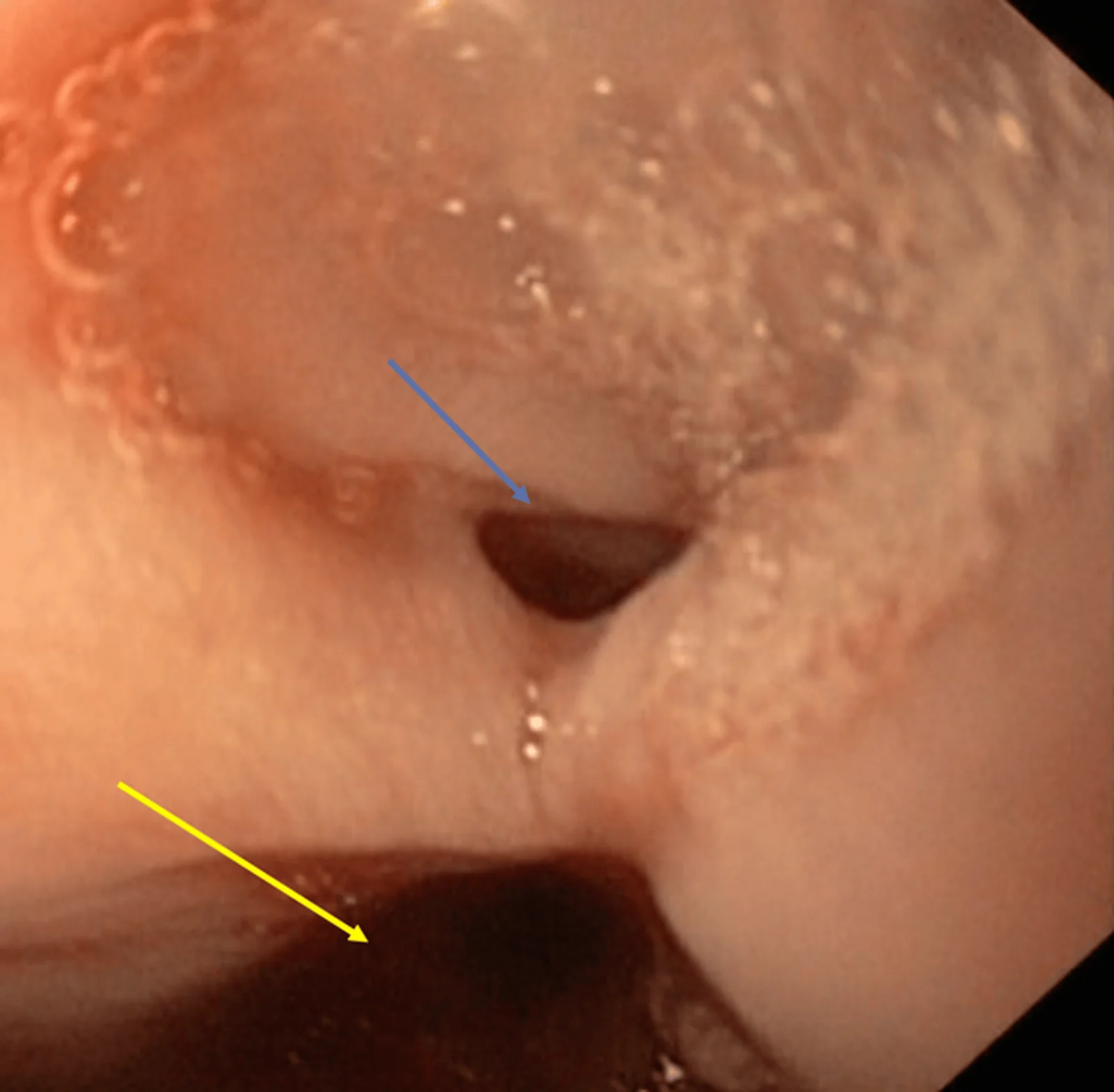
Endoscopic view of the upper oesophagus 21 cm from the incisors A double-lumen appearance is seen, with the native lumen inferiorly (yellow arrow) and an accessory orifice proximally (blue arrow), corresponding to the proximal communication of the duplication. The accessory orifice was intubated with a paediatric gastroscope and led to a tubular channel lined by oesophageal-type mucosa, running parallel to the native lumen and draining into the stomach, consistent with complete communicating tubular oesophageal duplication.

A second upper gastrointestinal endoscopy was performed with a paediatric gastroscope to clarify the luminal anatomy. The oesophagogastric junction was identified 35 cm from the incisors. At 21 cm, an accessory orifice was identified and intubated with the paediatric gastroscope; it led to a tubular channel lined by oesophageal-type mucosa that extended into the stomach (Figure 5). At 26 cm, the previously described inflammatory stricture was again encountered; it was traversable with the paediatric gastroscope and also led into the stomach. The gastric mucosa was macroscopically normal, and a 1 cm hiatus hernia was present. Los Angeles grade B reflux oesophagitis was observed, and the duodenum was normal.

Taken together, the endoscopic and radiological findings strongly supported a diagnosis of complete communicating tubular oesophageal duplication. Concomitant abnormalities comprised a mid-oesophageal stricture involving the native lumen, chronic dilatation of the native oesophagus, a hiatus hernia, gastro-oesophageal reflux, and intestinal metaplasia in biopsy specimens obtained from the native oesophageal stricture. Any pathophysiological relationships between the duplication and these abnormalities remain inferential. Initial management was conservative and included twice-daily proton pump inhibitor therapy, dietary and lifestyle measures, and clinical surveillance, with the aim of healing the inflammatory lesions before an interventional decision. At the two-month follow-up, dysphagia had markedly improved, and the patient tolerated a normal solid diet. No recurrent haematemesis was reported. His haemoglobin level was 13.4 g/dL, his body mass index was 22.2 kg/m², and treatment adherence was reported as good. Repeat endoscopy with targeted biopsies was planned to reassess the stricture and clarify the extent and significance of the intestinal metaplasia.

## Discussion

Communicating tubular oesophageal duplication is a rare malformation distinguished from cystic duplication by an elongated channel that may communicate with the native oesophageal lumen at one or more levels. The classic histological criteria for gastrointestinal duplication comprise contiguity with the digestive tract, a muscular wall, and a gastrointestinal mucosal lining. In unresected cases, however, the diagnosis usually rests on concordant endoscopic and radiological findings [1,3].

The distinctive features of this case are the complete and communicating nature of the duplication, two endoscopically traversable channels leading to the stomach, and presentation with haematemesis in an adolescent with dysphagia since childhood. The diagnostic delay was probably related to intermittent symptoms and the absence of nutritional consequences. Respiratory symptoms were absent, consistent with the absence of a tracheobronchial fistula on barium oesophagography and CT; this is clinically relevant because some communicating tubular duplications are associated with broncho-oesophageal fistulae or recurrent pulmonary infection [3].

Detailed anatomical assessment is essential. Endoscopy may reveal a stricture, accessory orifice, diverticulum-like appearance, or double lumen. Barium oesophagography is particularly useful for demonstrating communications, tracing the duplicated channel, and excluding a respiratory fistula. CT complements the evaluation by characterising mediastinal relationships, the content of the duplication, inflammatory changes, associated anomalies, and complications. Endoscopic ultrasonography may be considered in selected cases to assess wall structure and distinguish a duplication from a subepithelial lesion. Fine-needle puncture should be used cautiously when a cystic component is suspected because of the reported risk of infection [1,5].

The structure exhibited endoscopic and histological features of inflammation. We hypothesise that oesophageal stasis, documented reflux, and chronic irritation at the communication sites may have contributed to its development, although a causal relationship cannot be established. The precise source of haematemesis could not be identified. No active bleeding, blood, or stigmata of recent haemorrhage were observed endoscopically. Potential sources included the ulcerated stricture, reflux oesophagitis, and inflamed mucosa within the duplicated channel. The intestinal metaplasia on biopsy requires cautious interpretation because of its unusual location, active inflammation, and the duplicated anatomy. The original biopsy specimens were obtained exclusively from the ulcerated stricture in the native oesophageal lumen. Neither the cardia nor the duplicated channel was sampled. Although this location makes inadvertent cardia sampling or metaplasia within the duplicated channel unlikely, the absence of systematic endoscopic mapping precludes distinction between injury-related glandular or intestinal metaplasia and Barrett's oesophagus. Current guidance emphasises a standardised endoscopic description of the suspected segment and histological confirmation of intestinal metaplasia [6].

No specific therapeutic consensus exists for completely communicating tubular oesophageal duplication because the available evidence is limited to a small number of case reports. Recommendations favouring surgery for symptomatic or complicated duplications derive predominantly from cystic lesions, particularly those associated with compression, infection, bleeding, or concern for malignant transformation [1,2], and cannot necessarily be extrapolated to tubular variants. In completely communicating tubular forms, the shared wall and endoluminal communication may permit selected endoscopic approaches, including septotomy or fenestration, with favourable outcomes reported in several cases [7-9].

In the present case, several factors should guide management: the young age, the persistent dysphagia, the inflammatory stricture traversable only with a paediatric endoscope, the associated reflux, the presence of intestinal metaplasia, and the absence of respiratory symptoms. A purely conservative approach risks perpetuating stasis and inflammation, whereas any endoscopic or surgical procedure should be preceded by complete mapping of both lumina, their communications, and their mediastinal relationships. A multidisciplinary discussion involving interventional gastroenterologists, gastrointestinal or thoracic surgeons, radiologists, and pathologists, therefore, appears essential.

The main limitation of this report is the absence of histological confirmation of the duplicated wall because no resection had been performed at the time of writing. Nevertheless, the concordant findings on barium oesophagography, CT, and repeat endoscopy strongly support the diagnosis of communicating tubular oesophageal duplication. Clinical, endoscopic, and histological follow-up is required to document the course of the dysphagia, monitor the stricture, and clarify the significance of the intestinal metaplasia.

This case shares the delayed diagnosis associated with chronic dysphagia described in adolescents and young adults by Coumaros et al. and Familiari et al. [7,9]. Other forms may be diagnosed in adulthood or complicated by a broncho-oesophageal fistula, as reported by Kim et al. [3]. Typical endoscopic findings include an accessory orifice, double lumen, longitudinal septum, or traversable duplicated channel. Barium oesophagography and CT define the communications with the native oesophagus, the tubular extension, and the presence or absence of a respiratory fistula [3,4]. Reported management varies with anatomy and complications and includes surgery for complex or complicated forms and endoscopic septotomy or fenestration in selected cases [4,7-9]. In the present case, the inflammatory stricture and reflux oesophagitis justified initial mucosal healing before an endoscopic or surgical procedure was considered.

## Conclusions

Complete communicating tubular oesophageal duplication is an exceptional cause of chronic dysphagia. In a young patient, long-standing dysphagia associated with oesophageal dilatation, an inflammatory stricture, and an accessory endoscopic orifice should prompt a search for a duplicated oesophageal lumen. Diagnosis depends on complementary endoscopic and radiological assessment, while management should be individualised according to symptoms, anatomy, complications, and the feasibility of endoscopic or surgical treatment in an expert centre.
